# Supplementary material for: Identifying barriers and facilitators for health risk behaviours among people with severe mental illness in Bangladesh and Pakistan: a qualitative study
Source: Glob Ment Health (Camb). 2025 May 30;12:e63. doi: 10.1017/gmh.2025.10016 (PMC12231305; doi:10.1017/gmh.2025.10016)
Supplement: Nisa et al. supplementary material 2 — Nisa et al. supplementary material [file S2054425125100162sup002.pdf]

LIFT
Healthier life for people with SMI

Topic Guide participant with SMI

**Checklist for the interviewer**		
1.	Interviewee knows about research objectives and methodology	
2.	Interviewee knows about Information Sheet	
3.	Interviewee knows about being recorded	
4.	Informed consent	
5.	Private Space to conduct interview	

Notes about the topic guide and interview:

- The questions are a guide to facilitate the interview/discussion; there are prompts to help ask questions/get more information. If answers cover more than one question you do not need to ask the additional questions.
- Please select 2-3 photos from the provided options each (food, physical activity and sleep), depending on the participant's preference.
- The interviewee has the right to not answer any questions and stop the interview at any time.
- The interviewer must switch off their mobile phone.
- If you are worried about the mental or physical health of the interviewee please ensure you follow the outlined procedures.
-

The aim of data collection is to explore the research participants' diet, physical activity and sleep and the barriers and facilitators that they confront in their daily life routine. The statement about GDPR can be found at:

https://www.york.ac.uk/healthsciences/research/trials/trials-gdpr/
https://www.york.ac.uk/healthsciences/research/trials/trials-gdpr/research-partcipants/

Healthier life for people with SMI

Date:
Interviewer:
Interview Time: Start time: End time:

1. **<u>Introduction</u>**

Hello! How are you today? My name is ___________, and I am a research fellow at IOP and RMU. Thank you for agreeing to talk to me today. We would like to talk to you about your experiences. We are interested in your opinion and there are no wrong or right answers. You do not need to answer every question and let me know if anything is not clear. The interview will not be longer than an hour, and you can stop it at any time.

- Outline the aims and objectives of the research
  Researcher says: *(Referring to workshop and photo taking activity)*
  We are interested in hearing your views about diet, physical activity and sleep. As you know, this research is about people with severe mental illness, your doctor has diagnosed you and I completely understand how much your health is important for you. We would like to learn more about what helps and hinders you in having a healthy diet, doing physical activity and good sleep during your daily life.

- Explain what data will be used for and how it will be treated.
  Researcher says: *The collected data will be kept with the study team and the researchers will not share it with anyone else. Your confidentiality will be maintained and you will not be able to be identified in the reporting of the results of the research.*

Begin recording
With participants' permission, begin recording.

Contextual information
The interview should begin by asking participants to introduce themselves
Researcher says: *Now, Mr / Ms, I would like to know how did you arrive at IoP and how often do you visit the hospital? It is great that you take interest in your good health. Let's begin the discussion:*

Diet
First We would like to discuss your eating habits, both when it comes to healthy and unhealthy foods, as well as the factors that either hinder or help you in consuming these foods

Photo voice prompts (if they have taken photos related to diet)**:**
First let's take a look at one of the photos you took:
What is this photo showing?, What is really happening?)
Why did you take this photo?
How does it relate to your eating habits/ what you eat?
Do you think the food in this photo is healthy or unhealthy?
What does the photo show about what influences you to eat more or less healthily?
Why does this problem or strength exist?
What can we do about it?

(Repeat for other diet-related photos)

No.	Questions	Prompts
1.	Could you tell me what you eat on a "typical" day?	Breakfast, lunch, dinner, snacks, drinks.<br>Prompts if struggle to answer:<br>What did you eat yesterday?<br>Is that "typical"?<br>What did you eat that you normally do not?<br>What else would you normally eat?
2.	How healthy do you consider your typical daily diet? Please explain.	What food that you eat do you consider healthy?<br>What do you not consider healthy?<br>What could you be eating less of?<br>What could you eat more of?
3.	What food do you need to eat as part of a "healthy" diet? Why do you need this food? What makes it "healthy"?	Ask for examples<br>E.g Could discuss 2/3, could discuss types of food<br><br>What type of food do you consider healthy that you do not eat
4.	Would you like to have a healthier diet?	What benefits do you think it would have for you?
5.	What stops you from eating "healthy" food?	Taste, smell, cost, availability, quality, time, knowledge, personal motivation,

		encouragement from others<br>Discuss the examples given in question 3
6.	What encourages you to eat healthy food?	Taste, preparation, availability, advice from family/professionals<br>Discuss examples
7.	Can you tell me what foods you consider “unhealthy” that you eat? Why are they unhealthy?	Could discuss 2/3, could discuss types of food .<br>Prompt/extra question: what type of food do you consider unhealthy that you do not eat?
8.	Why do you eat “unhealthy” foods?	Taste, availability, cost, time constraints, social/family pressures.<br>Discuss examples
9.	Is there anything that stops you from eating unhealthy food/too much unhealthy food?	Knowledge, availability, cost, family members<br>Discuss examples
10.	Apart from the type of food itself is there anything else that is important in making what you eat ‘’healthy’’ or ‘’unhealthy’’	Amount, time of eating, way it makes you feel
11.	Is there anything you would suggest that may help you and/or other people to eat more ‘healthy’ food and less ‘unhealthy’ food?	Personal level, family/community level, health services, policy<br>If you have tried to eat more healthily before, what has helped you?
12.	Are there any other barriers for you or other people having a healthy diet?	Restricted access/ low motivation / purchasing power / quality / colour / inability to eat / taste / smell / other?
13.	Would you like to learn more about healthy diet? Who do you think should advise you on this/ give you this information?	
14.	Do you have anything else you would like to discuss or say about the type of food you eat, and having a healthy diet?	If you had to give one piece of advice to someone looking to improve their diet and eat more healthily, what would it be?

PHYSICAL ACTIVITY:

We would like to talk with you about the physical activity that you do and what helps you, or is a barrier to you being physically active.

Photo voice prompts (if they have taken photos related to physical activity)**:**
First let's take a look at one of the photos you took:
What is this photo showing?
How does it relate to your physical activity?
What does the photo show about what helps you to be physically active or challenges that you face?

How frequently do you engage in this type of physical activity?

(Repeat for other physical activity related photos)

Q No.	Questions	Prompts
15.	Could you tell me about the physical activity you currently do?	Sports/ exercise<br>Physical activity during daily life e.g. walking, cycling, taking the stairs, physical work, gardening.<br>What types of activities?<br>How often?
16.	Has the physical activity that you do changed over time?	What do you think has influenced that?<br>E.g. change in physical ability, mood, motivation, medication, time pressures, environment.
17.	Would you like to be more physically active?	What benefits do you think it would have for you?
18.	Why would you like to be more physically active	
19.	What is your favourite type of physical activity? Why?	How does it make you feel, physically and mentally?
20.	What motivates you to be physically active?	e.g. fitness, health, stress relief, enjoyment, need to carry out physical activity for work, travel, daily life?<br>How does physical activity contribute to your overall

		wellbeing/ quality of life?
21.	What do you think helps (or would help) you, or other people, to be physically active?	Access to facilities, preferred environment/ atmosphere, enjoyable activities, preferred time of day, cost, self-motivation (goal-setting/ routines), social support (from family/friend/carer or activity group).
22.	What do you think are barriers or challenges to you, or other people, being physically active?	Physical health issues, mood, motivation, confidence, knowledge, cost, access to facilities, time, social support.
23.	If you could make one change to improve your physical activity, what would it be?	What steps could you take to implement this change?

SLEEPING PATTERNS:
We would like to talk with you about your sleep and what helps, or makes it difficult for you to sleep well.

Photo voice prompts (if they have taken photos related to sleep):
First let's take a look at one of the photos you took:
What is this photo showing?
How does it relate to your sleep?
What does the photo show about what helps you to sleep well or challenges that you face? (Can you see anything in this photo that you think could help you sleep better or make your sleep more comfortable?)

(Repeat for other sleep related photos)

Q No.	Questions	Prompts
	In your opinion, what are good sleeping habits?	What are the benefits of getting a good sleep?
24.	Could you tell me about your usual sleep routine?	When do you usually go to sleep?<br>What time do you usually wake up?<br>Do you have a regular sleep routine or do you sleep at different times?<br>Is it different on work days/ days

		off?<br>Do you wake during the night? (How often, how long?)<br>Do you sleep during the day?
25.	Generally, do you think you sleep well or not very well?	How rested do you feel after a night's sleep?<br>How does this affect you during the day?
26.	Do you experience any particular sleep problems or disturbances?	E.g.Insomnia, sleep apnea, vivid dreams, or frequent awakenings.
27.	What do you think makes it difficult for you, or other people, to get a good night's sleep?	Medication/ Physical or mental health symptoms/ Stress/ Work/ Change in routine/ Sleep Environment (crowded, noisy, light, comfortable bedding?) /Caffeine/Diet/Electronic Devices
28.	How do you try to improve your sleep quality?	E.g. Bedtime routines, relaxation techniques, dietary changes.
29.	What do you think helps you, or other people, to get a good night's sleep?	Sleep arrangements/ environment/ regular routine/ relaxation/ daytime physical activity/ medication/ family or carer support.
30.	Have you ever consulted a healthcare professional or sleep specialist regarding your sleeping patterns or sleep-related issues?	What recommendations or advice did they provide?

Closing
Thank the participants for their time and participation.
Offer an opportunity for them to share any additional thoughts or concerns regarding barriers and facilitators for a healthy diet, physical activity and good sleeping patterns.

Topic Guide Caregiver

Introduction
The aim of data collection is to explore the diet, physical activity and sleep of the research participants with severe mental illness and the barriers and facilitators that they confront in their daily life routine. The statement about GDPR can be found at:

https://www.york.ac.uk/healthsciences/research/trials/trials-gdpr/
https://www.york.ac.uk/healthsciences/research/trials/trials-gdpr/research-partcipants/

A) Ground Rules

Participants can be instructed to switch off mobile phones. We want to hear from everyone. To ensure that each participant can fully express their thoughts and be heard, please speak one at a time.

- Rapport Building
  Researcher greets and introduces him/ herself to the carers: *Hello! How are you today? Thank you for agreeing to take the time to participate in this focus group, which will last for approximately 40 to 60 minutes.*

- Outline the aims and objectives of the research
  Researcher says: *(Referring to workshop and* photo taking *activity)*
  As you know, this research is about people with severe mental illness. I completely understand how much your relative's health is important to you. As you have taken part in the workshop activity related to photo taking, I would like to ask you a few questions. We want to hear your views about what helps and hinders people with severe mental illness having a healthy diet, being physically active and sleeping well..

- Explain what data will be used for and how it will be treated.
  Researcher says: *The collected data will be kept with the study team and researchers will not share it with anyone else. Your and the patient's confidentiality will be maintained and you will not be able to be identified in the reporting of the results of the research.*

- Explain the focus group will be recorded, with the participant's permission, so there is an accurate record of what is said.
  Researcher says: *The focus group will be audio-recorded. This will not be shared with anyone outside of the research team. Is that okay?*

Begin recording
With participants' permission, begin recording.

Contextual information
The focus group should begin by asking participants to briefly introduce themselves.

Researcher says: *(Now, Mr / Ms, I would like to know about you, kindly if you may introduce yourself? Could you please tell me your name and how long you have been a caregiver?*
Let's begin the discussion:

Diet
First we would like to talk about diet. We would like to talk with you about the person who you care for eating habits, and what helps and hinders them to eat healthily.

Category	No.	Questions	Prompts
Diet	**1.**	Can you describe what the person you care for eats on a typical day?	What foods do they like to eat?<br>When do they usually eat? (Regular mealtimes? Snacks?)
	**2.**	Who cooks their meals? Who shops for food?	Where do they usually buy food? Why?
	**3.**	In your opinion, what is a healthy diet?	What types of food do you think are healthy/ unhealthy?<br>Amounts<br>Routines<br>What are the benefits of eating a healthy diet?
	**4.**	What are the challenges for the person you care for/ people with severe mental illness eating a healthy diet?	What stops them from eating healthy foods?<br>What makes them eat unhealthy foods? Or too much/ too little food?<br><br>Taste, cost, availability, quality, time, knowledge, personal motivation, medication side effects, social/ cultural factors
	**5.**		

		What encourages or supports the person you care for/ people with severe mental illness to eat healthily?	Taste, preparation, availability, personal motivation, advice from family/ professionals, social/ cultural factors
	**6.**	Is there anything you would suggest that may help people with severe mental illness eat more 'healthy' food and less 'unhealthy' food?	Personal level, family/ community level, health services, policy
	**7.**	Do you think it would be helpful for you, or the person you care for, to learn more about healthy diet?	Who do you think should advise you on this/ give you this information?
**Physical activity:** We would like to talk with you about the physical activity that the person you care for does, and what helps, or is a barrier to them being physically active.			
Physical Activity	**8.**	Could you tell me about the physical activity that the person you care for currently does?	Sports/ exercise<br>Physical activity during daily life e.g. walking, cycling, taking the stairs, physical work, gardening.<br>What types of activities?<br>How often?
	**9.**	What do you think are the benefits of being physically active?	Do you think there are any particular benefits for people with severe mental illness?
	**10.**	What challenges or barriers do you think there are to the person you care for, or people with severe mental illness, being physically active?	Physical health issues, mood, motivation, confidence, medication side effects, knowledge, cost, access to facilities, time, social support.
	**11.**	What do you think helps the person you care for, or other people with severe mental illness, to be physically active?	*Access to facilities, preferred environment/ atmosphere, enjoyable activities, preferred time of day, cost, self-motivation (goal-setting/ routines), social support (from family/friend/carer or*

			activity group).
	**12.**	Is there anything you would suggest that could help people with severe mental illness to be more physically active?	Personal level, family/ community level, health services, policy
**SLEEPING PATTERNS:**<br>We would like to talk with you about how the person who you care for sleeps and what helps, or makes it difficult for them to sleep well.			
	**13.**	In your opinion, what are good sleeping habits?	What are the benefits of getting a good night's sleep?<br>Do you think there are particular benefits for people with SMI?
Slee p	**14.**	How does the person who you care for sleep?	When do you usually go to sleep/ wake up?<br>Regular sleep routine or varies?<br>Sleep problems/ disturbances?
	**15.**	What do you think can make it difficult for the person you care for, or other people with SMI, to get a good night's sleep?	Medication/ Physical or mental health symptoms/ Stress/ Work/ Change in routine/ Sleep environment (crowded, noisy, light, comfortable bedding?) /Caffeine/Diet/Electronic Devices
	**16.**	What do you think can help the person you care for, or other people with SMI, to get a good night's sleep?	Sleep arrangements/ environment/ regular routine/ relaxation/ daytime physical activity/ medication/ family or carer support.
	**17.**	Have you ever consulted a healthcare professional or sleep specialist regarding the sleeping patterns or sleep-related	What recommendations or advice did they provide?

		issues of the person you care for?	

Support and Resources
Discuss any support systems or resources that carers think are/ would be helpful for people with SMI in maintaining a healthy diet, being physically active, and maintaining good sleeping habits such as healthcare professionals, community programs, or online resources.

Future Goals and Recommendations
Ask for any suggestions or recommendations they have for improving support and resources in promoting healthy eating, physical activity and good sleep in people with SMI.

Closing
Thank the participants for their time and participation.
Offer an opportunity for them to share any additional thoughts or concerns regarding barriers and facilitators for a healthy diet, physical activity and good sleeping patterns.

LIFT
Healthier life for people with SMI

Topic Guide Healthcare provider

A) Introduction

The aim of data collection is to explore research participants' views about healthy diet, physical activity, and good sleep. Primarily, this research focuses on the barriers and facilitators that people living with SMI confront in their daily life routine. The statement about GDPR can be found at:

https://www.york.ac.uk/healthsciences/research/trials/trials-gdpr/
https://www.york.ac.uk/healthsciences/research/trials/trials-gdpr/research-partcipants/

B) Ground Rules

Participants can be instructed to switch off mobile phones or to hand over their mobile phones to the research assistant. We want to hear from everyone. To ensure that each participant can fully express their thoughts and be heard, please speak one at a time.

- Rapport Building
  Researcher greets: (*Hello! How are you today? Thank you for taking the time to participate in this focus group, it is much appreciated. The focus group will last for approximately 60 to 90 minutes.*

- Outline the aims and objectives of the research
  Researcher says: *As you know, this research is about severe mental illness, the participants we have at IoP are diagnosed and referred to the research team. I completely understand how important the health of your patients is to you. This research is to explore the barriers and facilitators that people with SMI experience in their daily lives, in having a healthy diet, doing physical activity and for good sleep.We would like to learn more about your views on this.*

- Explain what data will be used for and how it will be treated.
  Researcher says: *The collected data will be kept with the study team and the researchers will not share it with anyone else. Your confidentiality will be maintained and you will not be able to be identified in the reporting of the results of the research.*

- Explain the focus group will be recorded, with the participant's permission, so there is an accurate record of what is said.
  Researcher says: The focus group will be audio-recorded. This will not be shared with anyone outside of the research team. Is that okay?

Order of conversation

Psychiatrist-Psychologist-Nutritionist-Staff Nurse-Social Worker

Begin recording

With participants' permission, begin recording.

Contextual information

The focus group should begin by asking participants to introduce themselves.
Go with the flow of the conversation.

Participants will be given a background of the study.
They were presented with a case study as mentioned below:
This girl's name is Nadia. After this she was guided by her friend to visit the Institute of Psychiatry, where she was diagnosed as a case of Depression with Psychosis (SMI) and she is currently receiving treatment..

Question	Prompts
Do you see patients like Ms Nadia in your routine practice?<br>We will be discussing people like Nadia diagnosed with SMI	Which one of the SMI diagnoses is the most common?
In your experience, What do you think are the main issues Ms. Nadia may face with her diet?<br>In general, what are the dietary habits of people with SMI?<br>In your opinion, What are the most common barriers that people with SMI face when trying to maintain a healthy diet? How can we eliminate or reduce the barriers?	Meal choices, eating habits, behaviour related to diet<br>Whether this is same or different with people with different diagnoses?<br>What stops them from eating healthy foods?<br>What makes them eat unhealthy foods? Or too much/ too little food?<br>Taste, cost, availability, quality, time, knowledge, personal motivation, medication side effects, social/ cultural factors
What issues Ms. Nadia may face related to Physical activity? In general, how physically active do you think people with SMI are?<br>What challenges or barriers do you think hinder people with severe mental illness, being physically active?<br>How can we eliminate or reduce the barriers?	What types of activities do they do?<br>Sports/ exercise<br>Physical activity during daily life e.g. walking, cycling, taking the stairs, physical work, gardening.<br>Whether this differs with people with different diagnoses? In your experience, what are the most common barriers that people with SMI face when trying to maintain a healthy level of physical activity?
What sleep-related issues may Ms. Nadia face? In general, how well do you think people with SMI sleep? What do you think can make it difficult for your patients with SMI to get a good night's sleep?<br>How can we eliminate or reduce the barriers?	Regular/ irregular sleep routines?<br>What sleep problems/ disturbances do they encounter?<br>Whether this differs with people with different diagnoses? Medication/ Physical or mental health symptoms/ Stress/ Work/ Change in routine/ Sleep environment (crowded, noisy, light, comfortable bedding?) /Caffeine/Diet/Electronic Devices
What advice would you give Ms. Nadia?<br>Any different advice as per their type of SMI.	Anything that would help them with problems in their sleep, diet, physical activity
What can Nadia and people with SMI do to help themselves? How can they support themselves? Is there any plan of formulating a policy for good diet, sleep and physical activity.for this population?<br>Do you know any programs or policies promoting healthy habits for people with severe mental	Anything that could be useful to improve their lifestyle…<br>What key elements or strategies have contributed to the success of these initiatives?<br>Are there any best practices or lessons

illness, either in Pakistan or elsewhere? What insights can we gain from these experiences?<br>Do we have any healthcare policies in Pakistan that focus on the needs of individuals with severe mental illness, particularly in promoting a healthy lifestyle?<br>How do these policies impact the provision of nutrition, physical activity, and sleep support for individuals with SMI in your setting?"<br>What are the challenges or barriers you encounter in implementing these policies/guidelines effectively?	learned that could be applied to our efforts in promoting healthier lifestyles for individuals with SMI?<br>Policies/guidelines that concern physical health/reducing weight?How are these policies structured? What areas do they specifically address within your facility? Can you give specific examples? Are there any challenges or opportunities that have arisen due to these policies in your healthcare setting?<br>Can you share examples?
What are the most challenging aspects for Ms. Nadia?	Specifically, which of the following problems—having good sleep, maintaining a healthy diet, or engaging in regular physical activity—is most commonly experienced by those with SMI? and How they differ with diagnosis
**Facilitators**	
What encourages or supports your patients with severe mental illness to eat healthily?<br>What are the support systems, strategies or resources that your patients with SMI have found helpful in maintaining a healthy diet?<br>What would you suggest that may help people with severe mental illness eat more 'healthy' food and less 'unhealthy' food?	Taste, preparation, availability, personal motivation, advice from family/ professionals, social/ cultural factors<br>E.g. Healthcare professionals, community programs, or online resources. Ask for specific examples. What did they do? Do you know the outcome?<br>Personal level, family/ community level, health services, policy
What do you think helps your patients with severe mental illness to be physically active? Do you know of any support systems, strategies or resources that your patients with SMI have found helpful in supporting them to be physically active?<br>What would you suggest that could help your patients with severe mental illness to be more physically active?	Access to facilities, preferred environment/ atmosphere, enjoyable activities, preferred time of day, cost, self-motivation (goal-setting/ routines), social support (from family/friend/carer/group<br>E.g. Family & caregivers..Healthcare professionals, community programs, or online resources. Ask for specific examples. What did they do? Do you know the outcome?<br>Personal level, family/ community level, health services, policy
What do you think can help patients with SMI, to get a good night's sleep?<br>What are the support systems, strategies or resources that patients with SMI have found helpful in improving their sleep?	Sleep arrangements/ environment/ regular routine/ relaxation/ daytime physical activity/ medication/ family or carer support.<br>E.g. Healthcare professionals, community

	programs, or online resources.<br>Ask for specific examples<br>What did they do?<br>Do you know the outcome?
What would be helpful for the healthcare providers to help people with severe mental illness with their diet, sleep and physical activity?	

Future Goals and Recommendations

Ask for any suggestions or recommendations they have for improving support and resources in promoting healthy eating, physical activity and good sleep in people with SMI.

Closing

Thank the participants for their time and participation.

Offer an opportunity for them to share any additional thoughts or concerns regarding barriers and facilitators for a healthy diet, physical activity and good sleeping patterns.
